# Consistency of alerts generated by, and implementation of, the NHS England acute kidney injury detection algorithm in English laboratories

**DOI:** 10.1007/s40620-024-02030-6

**Published:** 2024-08-04

**Authors:** Ryan Aylward, Anna Casula, Nicki Tiffin, Yoav Ben-Shlomo, Brian Rayner, Kate Birnie, Fergus John Caskey

**Affiliations:** 1 Bristol Medical School, Population Health Sciences, University of Bristol, First Floor, 5 Tyndall Avenue, Bristol, BS8 1UD UK; 2 Division of Nephrology and Hypertension, Department of Medicine, Faculty of Health Sciences, University of Cape Town, Cape Town, South Africa; 3 UK Renal Registry, UK Kidney Association, Bristol, UK; 4 South African National Bioinformatics Institute, University of Western Cape, Cape Town, South Africa; 5 Southmead Hospital, NHS North Bristol Trust, Bristol, UK

**Keywords:** Acute kidney injury, Detection, Algorithm, Renal registry

## Abstract

**Background:**

National Health Services (NHS) England mandates that an acute kidney injury (AKI) detection algorithm be embedded in laboratories. We evaluated the implementation of the algorithm and the consistency of alerts submitted to the United Kingdom Renal Registry (UKRR).

**Methods:**

Code was developed to simulate the syntax of the AKI detection algorithm, executed on data from local laboratories submitted to the UKRR, including alerts and serum creatinine (SCr) results spanning 15 months before and after the alert submission. Acute kidney injury alerts were categorized into stages 0/1/2/3. Inter-rater agreement (Gwet’s AC1) was used to compare local and centrally derived alerts at individual laboratory and commercial laboratory information management system (LIMS) levels, penalizing extreme disagreements.

**Results:**

The analysis included 9,096,667 SCr results from 29 labs (475,634 patients; median age 72 years, 47% female) between algorithm activation and data extraction (September 30, 2020). Laboratories and the central simulation generated 1,579,633 and 1,646,850 non-zero AKI alerts, respectively. Agreement was high within known laboratory information management system providers (0.97–0.98) but varied across individual laboratories (overall range 0.17–0.98, 0.17–0.23 in three). Agreement tended to be lower (Gwet’s AC1 0.88) with the highest baseline SCr quartile (median 164 μmol/L).

**Conclusions:**

Overall, alerts submitted to the UKRR are a valid source of AKI surveillance but there are concerns about inconsistent laboratory practices, incomplete adoption of the NHSE algorithm code, alert suppression, and variable interpretation of guidelines. Future efforts should audit and support laboratories with low agreement rates, and explore reasons for lower agreement in individuals with pre-existing CKD.

**Graphical abstract:**

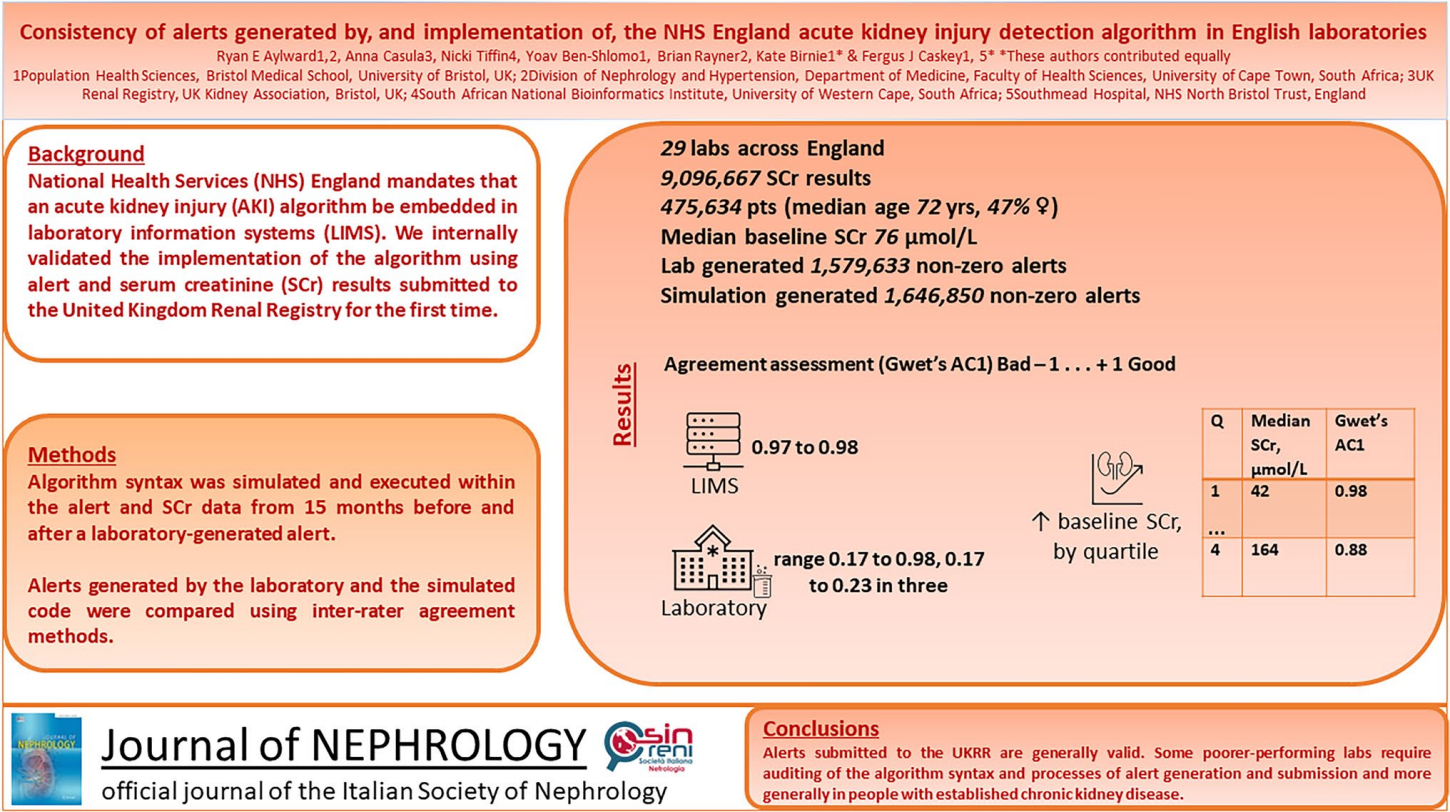

**Supplementary Information:**

The online version contains supplementary material available at 10.1007/s40620-024-02030-6.

## Introduction

National Health Service (NHS) England issued a Patient Safety Alert in 2014 (NHS/PSA/D/2014/010) to all pathology providers to standardise acute kidney injury (AKI) detection following the 2009 National Confidential Enquiry into Patient Outcome and Death which found that AKI was often preventable, missed and poorly managed [1, 2]. From March 2015, NHS England has required all 192 laboratories in England to integrate an algorithm to detect cases of AKI within its laboratory information management system (LIMS). Only 88% of surveyed English laboratories indicated that they were aware of such a mandate [3].

This falls under a wider ‘Think Kidneys’ initiative instituted by the Renal Association, now the UK Kidney Association, and United Kingdom Renal Registry (UKRR) which seeks to improve the quality of care and outcomes of patients with kidney disease (www.thinkkidneys.nhs.uk). Complementary resources to manage and prevent AKI were promoted under the second Patient Safety Alert issued in 2016.

Once a month, the laboratory sends these alerts, associated serum creatinine (SCr) results, and SCr results from the preceding 15 months to the UKRR [5]. Values for the subsequent 15 months after the alert are reported to the UKRR in the months following the alert. Alert and SCr data are linked by a unique Master Patient Index (MPI) using the pervasive NHS number.

The algorithm has previously been externally validated by comparing AKI detected by the algorithm with clinical diagnosis and nephrologist adjudication in the Grampian Laboratory Outcomes Morbidity and Mortality Study (GLOMMS) and found to be sensitive and specific [6, 7]. Although Think Kidneys guidelines were published, it is unknown how the algorithm syntax has been implemented in laboratory information management systems, and how laboratories process alerts. Variation in these processes and differences in patient populations, could contribute to disparities in AKI detection between laboratories. Using alert and SCr data submitted to the UKRR, the consistency of alerts received were evaluated in this study. This indirectly assesses firstly how the algorithm was translated into code and secondly how laboratories handle and submit alerts.

## Methods

### Study population

English laboratories that submitted high-completeness alert and pre- and post-alert SCr results from the initiation of the NHS England algorithm as part of the Tackling AKI study on 1 December, 2014 through 30 September, 2020 were included [8]. Data completeness is assessed at the point of UKRR alert receipt, the criteria of which are outlined in the UKRR 2020 annual AKI report [5].

Individuals aged < 18, and > 99 years were excluded because of concerns of incorrect recording of date of birth. Further exclusions were necessary based on missing/ irregular data and duplicates. The UKRR receives data about patients who start long-term dialysis for kidney failure—after which time AKI alerts are removed to avoid pre- and post-dialysis alerts.

### Central algorithm development

Local laboratory-generated alerts were compared with alerts derived using logic that simulated the NHS England algorithm (‘central code’) accessible at https://www.england.nhs.uk/akiprogramme/aki-algorithm/ and Supplementary methods S1. This central code was executed within the alert and pre- and post-alert SCr data received by the UKRR [2]. The central code was written by the first author using Stata MP version 16 (StataCorp, TX, USA). An index SCr at current testing was compared to historical baseline values for the same individual, if available, based on KDIGO creatinine criteria [2, 4]. The NHS England algorithm deviates from KDIGO criteria in that the look-back period is extended to 365 days [2]. The central code was executed in the alert and before/after SCr data, described above, to generate ‘central alerts’. Central AKI 0, 1, 2 and 3 alerts were created. The date of the first-ever received alert was taken as the date on which the algorithm went live within that laboratory. Local laboratory alerts are received as AKI 1,2 or 3. AKI 0 alerts were inferred centrally if no alert was received for that particular SCr value. Only SCr values that occurred after the live date, and not associated with an AKI alert, were recoded as AKI 0 because the algorithm had to have been active to theoretically fire an alert, if appropriate.

### Analysis

Descriptive statistics were used to summarise the baseline characteristics of participants and AKI alerts. Laboratory- and centrally-coded alerts were compared pair-wise for each SCr result using inter-rater agreement methods (Gwet’s agreement coefficient 1 [AC1]) [9–11]. Agreement coefficient magnitude, which ranges from − 1.00 to + 1.00, was interpreted as recommended by Landis and Koch [12]. ‘Substantial’ and ‘almost perfect’, that is 0.61–1.00, were deemed to be acceptable. Ordinal weighting, given in the Supplementary Table S1, was applied since AKI stages are not binary and are ordered by severity. Thus, partial agreement was still possible, but disagreement was penalized.

Agreement was also evaluated across commercial laboratory information management systems. Theoretically, there are potentially two versions of code: firstly, that which is encoded by the laboratory information management system provider and secondly, further modified by the laboratory. Agreement was thus examined for individual laboratories primarily.

### Exploratory analyses

Agreement was recalculated in several exploratory analyses to test various assumptions. These are outlined below.

*Sensitivity analysis*: Firstly, SCr values not associated with a laboratory alert were not recoded to AKI 0 as there might have been other reasons why an alert was not received (such as alert suppression or incomplete data submission). These laboratory alerts were, therefore, set to missing and were discarded from the complete-case analysis. Lastly, agreement was assessed for laboratories that submitted only complete SCr data as there was concern that missing SCr data might conflate the observed results as the central code was unable to ‘see’ all SCr data. Data were only designated complete if all months had non-missing alert and SCr data. The laboratory’s data were otherwise removed. This was necessary as only alert data, but not SCr data, are rated for completeness by the UKRR.

Sub-group analysis: agreement was assessed over quintiles of age and baseline SCr, and per calendar year.

## Results

### Baseline characteristics of the alerts included in this analysis

Thirty seven out of the 187 laboratories were included. Out of 1,966,003 AKI alerts representing 532,884 patients received by the UKRR from the beginning of the NHS England algorithm deployment from December 2014 to the end of September 2020, 1,579,663 alerts—from 475,634 individuals—were available for analysis; exclusions are shown in Fig. S1. Out of the 37 laboratories considered to have high data completeness, eight were excluded for other reasons. Two laboratories inconsistently sent monthly data leading to frequent missing SCr values over time. There was evidence of missing pre-alert SCr data for five laboratories. One laboratory was excluded because only SCr data were sent but not any alerts. Patient, alert and SCr characteristics are shown in Table 1. Laboratories included in this analysis were well represented across England (Fig. S2).

**Table 1:** Characteristics of alerts that were included in this analysis

	Local laboratory alerts, *N* = 1,579,663	Central alerts, *N* = 1,646,850
N persons	475,634	475,634
Age in y, median (IQR)	72.4 (59.6; 82.0)	
Sex, %		
Female	47%	
Nonzero alerts		
The median number of alerts per person (IQR)	3 (1; 8)	4 (2; 9)
Range	1; 252	1; 352
AKI 1, N (%)	988,284 (63%)	1,067,239 (65%)
AKI 2, N (%)	290,998 (18%)	349,405 (21%)
AKI 3, N (%)	300,351 (19%)	230,206 (14%)
Median creatinine at alert (IQR) μmol/L		
All AKI	164 (111; 264)	169 (111; 286)
AKI 1	132 (96; 187)	140 (97; 219)
AKI 2	181 (137; 237)	197 (139; 286)
AKI 3	414 (307; 555)	361 (238; 577)
Baseline SCr for the 1st alert^a^	77 (57; 108)	–
Reference Value 1^b^	–	83 (IQR 56; 132)
Reference Value 2^c^	–	87 (IQR 64; 127)
After the last alert	83 (60; 121)	–
Before and after SCr results		
N before (the 1st alert)	3,583,049	
Median number per person	17 (9; 13)	
N after (the last alert)	2,025,767	
Median number per person	13 (6; 26)	

*IQR* interquartile range, *AKI* acute kidney injury, *N* number, *SCr* serum creatinine

^a^Computed for the first alert here, but baseline SCr values are not received by the UKRR, ^b^ RV1, reference value (lowest baseline SCr) for preceding 0–7 days, only available for central alerts; ^*c*^RV2, reference value (median baseline SCr) for preceding 8–365 days, only available for central alerts

### Characteristics of the centrally-derived alerts

The central code computed a greater number of AKI non-zero alerts (1,646,850) than those received by the UKRR, indicating possible under-ascertainment or incomplete submission of alerts. Compared to local laboratory-generated alerts displayed in Table 1, the SCr at central alert was higher than local laboratory-generated alerts for AKI 1 (median 140 vs 132 μmol/L) and AKI 2 (median 197 vs 181 μmol/L) but was 53 μmol/L lower for AKI 3 (median 361 vs 414 μmol/L).

Reference Value 1, which could potentially be drawn during the prodromal illness before AKI is recognised, was lower than Reference Value 2 for AKI 0 (78 vs 86 μmol/L), but higher than Reference Value 2 for all other stages—AKI 1: 109 vs 90, AKI 2: 122 vs 89, and AKI 3: 161 vs 88 μmol/L. The index creatinine (C1): Reference Value ratio, which is used to calculate relative changes in SCr, averaged 1.62 (IQR 1.53; 1.75) for AKI 1, 2.30 (IQR 2.12; 2.56) for AKI 2 and 3.81 (IQR 3.21; 5.13) for AKI 3. Although the baseline (Reference Value 1) was higher, the median index creatinine: Reference Value ratio for AKI 1 from between 0 and 7 days was < 1.5 suggesting that the relative change in SCr that flagged AKI 1 was from the 8–365-day period or an absolute increase in SCr of > 26 μmol/L in 48 h.

### Local laboratory versus central generated alerts

Table 2 shows a cross-tabulation of local laboratory-generated alerts compared with those derived centrally. The local laboratory and central alerts mostly concurred. The majority were AKI 0.

**Table 2:** Matrix of raw numbers of laboratory versus centrally derived alerts

	Centrally generated alerts				
	0	1	2	3	Total
Local lab generated alerts after the first ever alert					
0					
Percent	**79.81**	1.82	0.51	0.49	82.64
Frequency	**7,260,258**	165,802	46,541	44,433	7,517,034
1					
Percent	1.85	**8.67**	0.23	0.11	10.86
Frequency	167,922	**788,731**	21,288	10,343	988,284
2					
Percent	0.33	0.26	**2.52**	0.09	3.20
Frequency	30,252	23,711	**229,065**	7,970	290,998
3					
Percent	0.34	0.70	0.50	**1.77**	3.30
Frequency	30,522	63,430	45,053	**161,346**	300,351
Total					
Percent	82.33	11.45	3.76	2.46	**100.00**
Frequency	7,488,954	1,041,674	341,947	224,092	**9,096,667**

The frequency and percent of alerts in perfect agreement are highlighted in bold

Laboratory 0 alerts were assumed on the basis that an alert was not generated for a given SCr value. This was only assumed for alerts generated after the first-ever alert for that laboratory was submitted as a proxy for the date of activation of the algorithm. Note that the number of alerts here reflects each individual SCr result available for analysis, hence the very many 0 AKI alerts.

### Inter-rater analysis of local laboratory versus central alerts

In general, agreement was almost perfect—overall Gwet’s AC1 was 0.98 and 26 laboratories computed a Gwet’s AC1 of > 0.80 (Table 3). Some alerts generated within laboratories showed only slight agreement with the central code; Gwet’s AC1 ranged from 0.17 to 0.23 in three. This was driven by two patterns of misclassification in these three laboratories: (1) central AKI 0 was misclassified as laboratory AKI 3 in 38–55% of central AKI 0 alerts and (2) central AKI 0 was misclassified as laboratory AKI 1 in 88–92% of central AKI 0 alerts.

**Table 3:** Agreement coefficients for anonymised individual laboratories

N lab alerts	N central alerts	Percent positive agreement	Gwet’s AC1 agreement	95% CI	LIMS provider
18,912	5,589	0.6852	0.1697	0.1564, 0.1830	LIMS1
42,262	15,789	0.6992	0.1726	0.1638, 0.1814	LIMS1
35,318	9,869	0.7146	0.2261	0.2172, 0.2351	Unknown
9126	5,438	0.9176	0.8518	0.8464, 0.8573	Unknown
118,433	91,156	0.9542	0.9241	0.9233, 0.9250	Unknown
81,974	65,234	0.9571	0.9428	0.9422, 0.9434	Unknown
54,069	68,744	0.9619	0.9460	0.9452, 0.9468	Unknown
21,522	29,385	0.9631	0.9497	0.9486, 0.9508	Unknown
45,720	31,098	0.9650	0.9521	0.9514, 0.9529	LIMS2
7976	8,891	0.9737	0.9613	0.9595, 0.9631	LIMS1
87,505	104,631	0.9733	0.9631	0.9626, 0.9636	LIMS2
82,902	95,496	0.9772	0.9662	0.9657, 0.9667	LIMS1
42,813	51,575	0.9791	0.9677	0.9669, 0.9685	Unknown
84,134	84,948	0.9766	0.9682	0.9678, 0.9686	Unknown
25,552	24,729	0.9809	0.9702	0.9693, 0.9711	Unknown
4408	8,232	0.9761	0.9702	0.9690, 0.9713	Unknown
75,392	102,496	0.9760	0.9703	0.9700, 0.9707	LIMS3
121,016	165,896	0.9806	0.9750	0.9747, 0.9753	Unknown
58,138	65,579	0.9826	0.9765	0.9761, 0.9769	LIMS1
37,312	41,825	0.9853	0.9793	0.9787, 0.9799	LIMS1
118,612	133,778	0.9852	0.9803	0.9800, 0.9805	LIMS2
26,784	30,183	0.9865	0.9808	0.9801, 0.9815	LIMS1
53,376	56,986	0.9902	0.9865	0.9862, 0.9868	LIMS2
110,469	118,988	0.9909	0.9866	0.9864, 0.9869	Unknown
26,617	27,624	0.9910	0.9879	0.9874, 0.9883	LIMS2
99,768	105,677	0.9916	0.9882	0.9880, 0.9884	Unknown
38,871	42,990	0.9938	0.9917	0.9914, 0.9919	Unknown
33,153	35,631	0.9943	0.9926	0.9923, 0.9928	Unknown
17,499	18,393	0.9959	0.9943	0.9939, 0.9946	Unknown

Sorted in ascending order of agreement. Coefficients are displayed to 4 decimal places as the CI was very narrow. The proprietary names of LIMS providers have been anonymised

*Lab* laboratory, *LIMS* laboratory information management system, *AC* agreement coefficient, *CI* confidence interval; *N* number

Table S2 shows that assessment of agreement was also almost perfect (> 0.80) across all three laboratory information management system providers; Gwet’s AC1 ranged from 0.97 to 0.98. The laboratory information management system provider was unknown for 16/29 laboratories. As such, the provider was only available for 43% of local laboratory alerts. Of the three laboratories demonstrating slight agreement, two of these utilised the laboratory information management system 1 provider, previously shown to be performing well at the laboratory information management system-level. This suggests a local laboratory rather than a laboratory information management system inconsistency. Cumulatively, these represented a small proportion of the total alerts (8%).

### Exploratory analyses

Firstly, given that there were laboratories that submitted incomplete alert data, these 11 laboratories were excluded. Table S3 showed that by excluding laboratories with patchy missing data, agreement was no higher (Gwet’s AC1 was 0.97).

The UKRR does not receive AKI 0 alerts and so local laboratory-generated alerts were not exactly comparable with alerts generated by the central code. For this reason, AKI 0 alerts were assumed on the basis that an alert was *not* received. Agreement, between nonzero AKI 1/2/3 alerts only, was once again almost perfect, though substantially lower, when local laboratory alerts were not recoded to AKI 0 – Gwet’s AC1 was 0.83 versus 0.97. Alert agreement is compared in Table S4 (number of alerts) and Table S5 (agreement coefficients).

An exploratory analysis, presented in Table S6, found that Gwet’s AC1, per calendar year, from 2015 to September 2020 was similar even during the 2020 Coronavirus-2019 pandemic (0.96 compared to 0.97 the previous year): laboratory implementation of the NHSE algorithm was sequential and the syntax was not altered over time. Serum creatinine data submission was incomplete for seven laboratories in 2020 compared to one in 2019.

Over quartiles of baseline SCr, agreement was high at lower SCr but decreased substantially at higher baseline SCr values. Gwet’s AC1 was 0.98 for quartile 1 but dropped to 0.88 for quartile 4 as shown in Table S7. Moreover, for the highest baseline SCr, quartile 4, the frequency of AKI 1 was greatest for central compared to local laboratory-generated alerts (18% [central] versus 15% [local]) but lower for stage 3 alerts (4% [central] versus 9% [local]). This suggests that severe AKI might be under-reported in people with pre-existing CKD because alerts are suppressed by laboratories.

Finally, agreement was found to be almost perfect (> 0.80) and consistent in younger and older people. In Table S8, the median age for each quintile ranged from 45 to 88 years and Gwet’s AC1 was between 0.81 – 0.85.

## Discussion

This study used routinely collected AKI alert and SCr data submitted to the UKRR, for the first time, to internally validate the NHS England AKI detection algorithm by assessing its implementation and consistency of AKI alerts generated by laboratories in England. Local laboratory alerts submitted to the UKRR were compared to alerts produced by simulated code. There was almost perfect agreement between local laboratory and centrally derived alerts, although implementation of the algorithm may not have been consistent in some individual laboratories and when the baseline SCr was high. This study was important since these alerts are used by the UKRR to report on AKI rates across England and researchers use these data to study various aspects of AKI care and outcomes [8, 13, 14].

Because the NHSE algorithm syntax was not provided, the code integrated into laboratory information management systems will not be identical. In this analysis, agreement was similar and almost perfect within three laboratory information management system providers, although not all providers were known. This is encouraging since it indicates compliance with the prescribed guidance. It also suggests that any divergence in agreement found is likely due to individual laboratory implementation or failed data submission. Nevertheless, it should not be surprising that there might be misinterpretation of the algorithm by laboratories given that even nephrologists do not always agree about how KDIGO criteria should be clinically applied in database research [16].

On the other hand, there was poor agreement within some individual laboratories. The three laboratories with the lowest agreement demonstrated high-quality data quality, defined by the UKRR as being highly non-missing, and implemented the algorithm in their laboratory information management systems in early 2016 and 2017. As well as having implications for clinical care, this has consequences for the UKRR which reports AKI incidence rates. In the UKRR 2018 AKI report, the sex- and age- adjusted rate varied from 2600 to 20,600 per million population [5]. The disagreement amongst a few laboratories in the present analysis might explain this variation although these three laboratories were not at the extreme incidences reported by the UKRR. Sawhney et al. have previously demonstrated that when similar methodological rules and age-sex adjustments are applied, there are usually few true differences in regional AKI incidence in the UK [15].

Some laboratories submitted incomplete data. Either there was missing information for certain variables or alert or SCr data were missing completely for one or more month(s). Agreement was very similar for laboratories contributing non-missing monthly data, suggesting that the effect of missing monthly data is negligible. The UKRR does attempt to reconcile demographic data with NHS Digital which might help to limit missing and remedy inaccurate records by cross-referencing and filling in missing information submitted to the UKRR with information held by NHS Digital.

At the laboratory level, those surveyed in a recent national audit indicated that it is not always the NHSE algorithm that is embedded (at least 11% admitted that the algorithm used was ‘in-house’) and not all SCr results are fed into the algorithm depending on the level of care that the SCr originated [3]. Also, alert calculation does not always occur in the laboratory information management system but instead in separate software [3]. Although the NHS England algorithm is automated, alert and SCr data submission to the UKRR is manual so it is impossible to estimate the extent of missing data which are also difficult to recover, beyond sending regular reminders to laboratories. Automated data transmission processes may overcome this obstacle. Only 56% of surveyed laboratories had a system in place to ensure that the data extracted from the laboratory information management systems and submitted to the UKRR were complete [3].

*Think Kidneys* permits suppression of alerts in certain circumstances such as alerts originating from neonatal and renal units. In up to 24% of laboratories, alerts from clinical disciplines and areas are also suppressed in practice: paediatrics, outpatient departments (OPDs), renal wards and outpatient departments, primary care and intensive care units. Laboratories often exclude alerts from renal unit location codes thereby excluding patients on dialysis but also potentially excluding patients with CKD, who are at increased risk of developing acute-on-chronic kidney disease, not yet requiring kidney replacement therapy [3].

Also, their best practice guideline notes that: *“The effect of previous AKI episodes on the diagnosis of new AKI episodes”* and *“Increases of serum creatinine over 48 h of more than 26 µmol/L (but less than 50% above baseline) in stable chronic kidney disease (CKD)”* should be addressed to reduce false positive alerts [5]. Laboratory information management systems and laboratories might adopt potentially incongruent code modifications as no specific advice is given. This is evidenced by the fact that only 75% of surveyed laboratories received the algorithm code from their laboratory information management system provider and 50% admitted that the code can be edited within their system which is concerning for non-standardisation of embedded code [3]. The UKRR confirms that no amendments to the original algorithm code have been made. Alternatively, the code may be similar, but alerts following the first alert, at which AKI is detected for the first time, may be suppressed and not sent to the UKRR. This might explain why agreement was very low for some and high for other laboratories which should be employing equivalent code and serviced by the same laboratory information management system provider.

‘Exception’ reports should be made compulsory and forwarded to the UKRR so that they may be scrutinised. Rules should be standardised in the *Think Kidneys* guideline to ensure laboratories are implementing comparable code. Although not recommended by the guideline, 87% of surveyed laboratories admitted that AKI alerts from obstetric patients were suppressed [3]. Box 1 gives a list of recommendations based on findings of this analysis.

Box 1 Recommendations to address noncompliance and standardise the implementation of the NHSE algorithm, alert processing and alert suppression

1. Laboratories must adhere to the NHS PSA 2014 mandating installation of the NHSE algorithm [2, 3].
2. A global stakeholder meeting including all LIMS providers and representatives from all laboratories should be held to understand reasons for noncompliance with the PSA and barriers to algorithm implementation and alert and creatinine result submission.
3. The *Think Kidneys* guidelines should be updated to include:
  - Example algorithm code to be embedded,
  - Clarification of where the algorithm should sit (LIMS only),
  - Which alerts to suppress (such as from dialysis patients) and how to achieve this with high specificity and sensitivity,
  - A list of suppressed alerts and reasons for suppression should be reported to the UKRR, and
  - A list of groups of patients who should not be suppressed (those with non-KRT CKD and admitted to intensive care).
4. A quality assurance initiative to ensure compliance with the guideline and standardisation.

Abbreviations: *NHSE* national health services England, *PSA* patient safety alert, *UKRR* United Kingdom renal registry, *LIMS* laboratory information management system, *CKD* chronic kidney disease, *KRT* kidney replacement therapy.

In this analysis, it was found that agreement fell at high baseline SCr values indicating possible misclassification of AKI as worsening CKD. Previous external validation of the algorithm found that up to 14% of CKD was misclassified as AKI when compared to nephrologist adjudication [7]. False positive AKI has previously been found to be more common in people with higher baseline SCr (133 μmol/L [> 1.5 mg/dL]) particularly because a 26.5 μmol/L (0.3 mg/dL) absolute increase, as included in defining KDIGO stage 1 AKI, is easier to reach at high SCr values when biological and analytical variation in SCr is higher [17, 18]. Future iterations of the NHS England algorithm might require redesign for use in people with CKD and there should be clarification of how alerts generated by people with CKD are processed within laboratory information management systems.

The findings of this study have important implications for other algorithm iterations and settings; broadly highlighting that AKI alert systems do not end with alert generation. Studies of alert algorithms need to be evaluated along the entire pathway from alert generation, processing, submission to a database and handling within the database. Algorithm validity studies often focus on the accuracy of the AKI detection rules but ignore the equally important components of practical algorithm implementation especially when algorithms are not centralised to a single laboratory information management system, laboratory, facility or electronic health record platform. Clearly laboratories have incongruent practices that are either based on interpretations of guidelines or adopted independent of specific guidelines. Guideline iterations should give very clear instructions about how the algorithm should be embedded within laboratory information management systems and alerts processed. Quality assurance initiatives are equally important to ensure compliance with the mandate and specific guidelines. Although this analysis evaluated a specific algorithm in a particular country, learnings about how to practically implement an algorithm, quality control alert generation and processing, and develop unambiguous and specific guidelines are transferable to other versions of algorithm code and settings of algorithm deployment. Also, more generally, KDIGO criteria to detect AKI may operate differently in community and hospital-based settings, necessitating consideration of the setting in which the SCr test was taken [19].

This study captured a substantial number of alerts and individuals over an extended period. The included laboratories were geographically representative of all in England. There were, however, some limitations as well. The dataset available for this analysis was a subset of relatively complete data from laboratories regularly and consistently sending files to the UKRR. An advantage of such is that it to some extent limits the problem of missing data—although there was concern for alert suppression. Future analysis of all 187 laboratories, after recovery of non-submitted alert and SCr files, would be important to validate the high agreement found in the current analysis. The UKRR does not receive any clinical information attached to the alert or SCr result so it was not possible to explore the possibility of confounding by clinical factors. Systematic bias is unlikely since the algorithm and processes are automated and agnostic to the clinical context. This study used routinely collected data that notoriously are subjected to missing data issues and duplicate entries, and as highlighted, the algorithm and the UKRR do not always receive all results. The UKRR, however, performs extensive data cleaning, reconciliation, and clarification with NHS Digital and communicates with laboratories directly to resolve irregularities. Unfortunately, it was not possible to evaluate the impact of alert (dis)-agreement on patient and clinician important outcomes due to the unavailability of such information, but given the high overall agreement, differences are likely negligible.

## Conclusion

The findings of this analysis would suggest that the AKI alerts that are submitted to the UKRR remain a valid source of AKI surveillance. Alert generation locally, rather than centrally at the UKRR, is acceptable although as highlighted, there are concerns for incongruent laboratory practices, incomplete adoption of the NHSE algorithm, alert suppression and variable interpretation of the Think Kidneys guidelines. Efforts should now focus on supporting laboratories with low agreement rates, understanding the clinical implications of, and reasons for, lower agreement rates in individuals with pre-existing CKD, and auditing laboratory practices.

## Supplementary Information

Below is the link to the electronic supplementary material.

Supplementary file1 (DOCX 752 KB)

## Data Availability

These data are available at reasonable request from the UKRR after consideration by the Data Release Group.
