# Supplementary material for: Consistency of alerts generated by, and implementation of, the NHS England acute kidney injury detection algorithm in English laboratories
Source: J Nephrol. 2024 Aug 4;37(8):2317–25. doi: 10.1007/s40620-024-02030-6 (PMC11649820; doi:10.1007/s40620-024-02030-6)
Supplement: Supplementary file 1 — Supplementary file1 (DOCX 752 KB) [file 40620_2024_2030_MOESM1_ESM.docx]

**Consistency of alerts generated by, and implementation of, the NHS England acute kidney injury detection algorithm in English laboratories**

Supplementary material S1

**Supplementary methods**

*Detailed description of the NHS England AKI detection algorithm*

A flowchart of the algorithm may be found at <https://www.england.nhs.uk/akiprogramme/aki-algorithm/>. If a previous SCr result exists in the 365 days prior to the index creatinine (called ‘C1’), the lowest value is used as the reference baseline if the result is within 0 – 7 days (‘RV1’) or the median of creatinine values within 8 – 365 days (‘RV2’). This reference value (RV) is compared to C1. The highest ratio is preferred. If the ratio (C1/RV) is < 1.5, and the difference between values within 48 hours is < 26 μmol/L, no alert (called an ‘AKI warning test’) is generated. Otherwise, a ratio ≥1.5 (or absolute value > 26 μmol/L; KDIGO 1), ≥2.0 but < 3.0 (KDIGO 2) or ≥3.0 (or absolute result > 354 μmol/L; KDIGO 3) generates an AKI alert[1].

If no prior SCr result can be found in the LIMS (which is limited to that laboratory; SCr results from other laboratories cannot be ‘seen’), C1 is compared to the laboratory reference interval, and if high, flagged as possible AKI or CKD with a recommendation to repeat testing. The UKRR does not receive these warning flags. The population reference interval may not be the same across all laboratories.

The algorithm discontinues generating an alert once these criteria are not met, but there is no definition of recovery included in the algorithm to delineate one AKI episode from another. Multiple alerts are generated per patient depending on how many SCr tests are requested (sometimes multiple per day) such that each patient may have multiple alerts during the same AKI episode. The UKRR does not receive the reason for the alert i.e., the threshold reached, or the specific RV (RV1 or RV2) used to generate the alert although pre-alert data are available[1].

Stata do file for the centrally written code is available at GitHub Repository: <https://github.com/RyAylwd/NHSEnglandAKIalgorithm>.

*Ordinal weights applied to penalize disagreement between the laboratory generated alerts and those derived centrally using simulated code.*

|  |  | **Central coded AKI** | | | |
| --- | --- | --- | --- | --- | --- |
|  |  | **0** | **1** | **2** | **3** |
| **Laboratory generated AKI** | **0** | 1.00 | 0.83 | 0.50 | 0.00 |
|  | **1** | 0.83 | 1.00 | 0.83 | 0.50 |
|  | **2** | 0.50 | 0.83 | 1.00 | 0.83 |
|  | **3** | 0.00 | 0.50 | 0.83 | 1.00 |

**Table S1**. These weights were derived from the kappaetc Stata user-written command.

**References**

1. Selby NM, Hill R, Fluck RJ. Standardizing the Early Identification of Acute Kidney Injury: The NHS England National Patient Safety Alert. Nephron. 2015;131(2):113–7.

**Supplementary results**


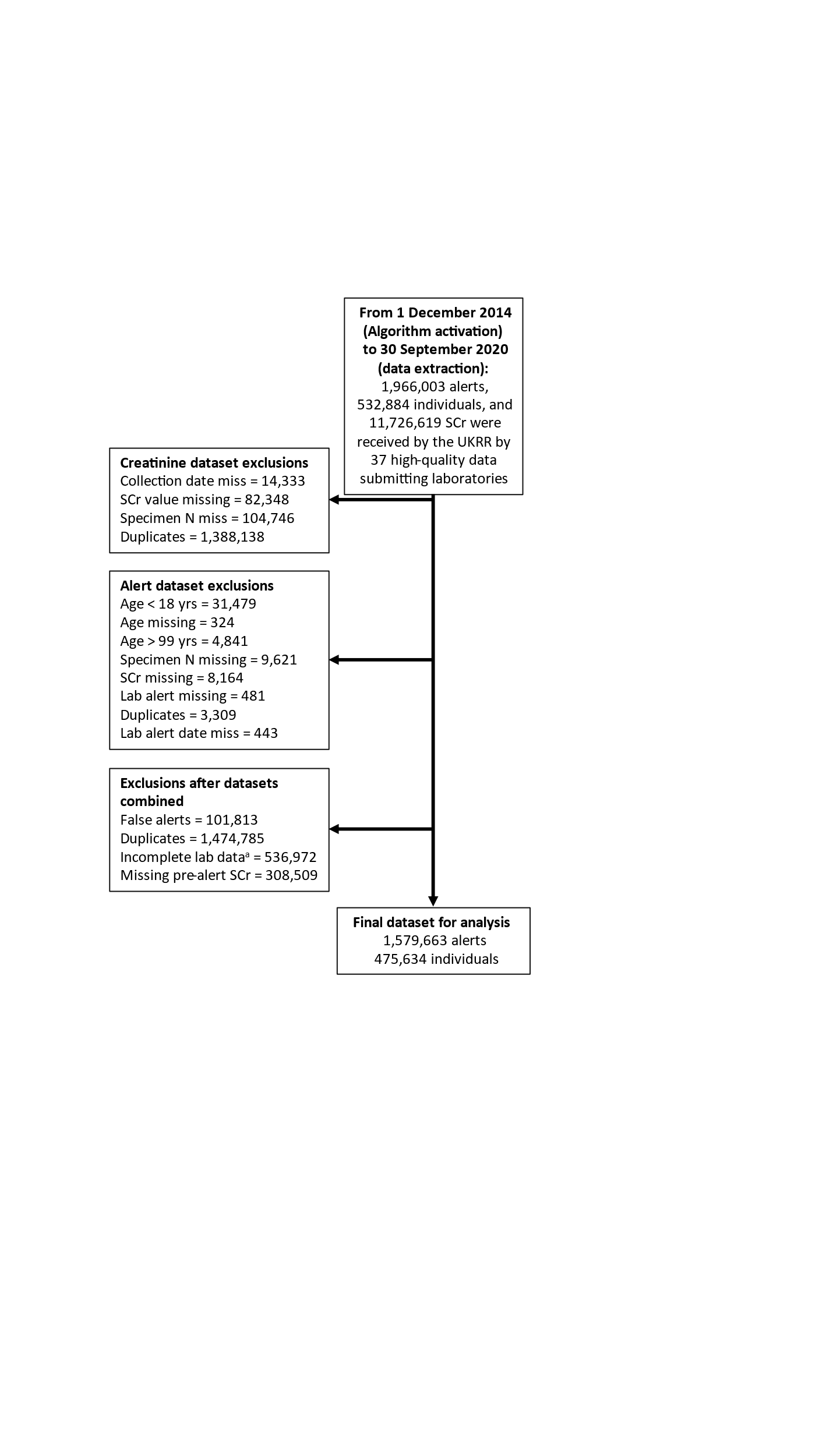


**Fig. S1.** Number of patients, alerts and serum creatinine data included in this analysis.

Alert and SCr datasets are received separately by the UKRR. For the alert dataset, each line of data was an alert. The SCr dataset was provided as one line per SCr result. Exclusions therefore refer to the number of alerts or SCr results, as appropriate. Datasets were first cleaned and then re-cleaned after appending together. False alerts appeared to occur before a baseline SCr was available, indicating that some SCr values were not submitted or were otherwise unusable because of associated missing data. These were recoded to missing rather than deleted to avoid losing the associated SCr result. For duplicates between alert and SCr datasets, the duplicate occurring from the SCr dataset was dropped and the duplicate from the alert dataset was retained. Only the age < 18 years exclusion was pre-defined. Numbers do not exactly total as exclusions were dropped sequentially. ^a^ See text for details. Abbreviations: SCr, serum creatinine; lab, laboratory; miss, missing; N, number.


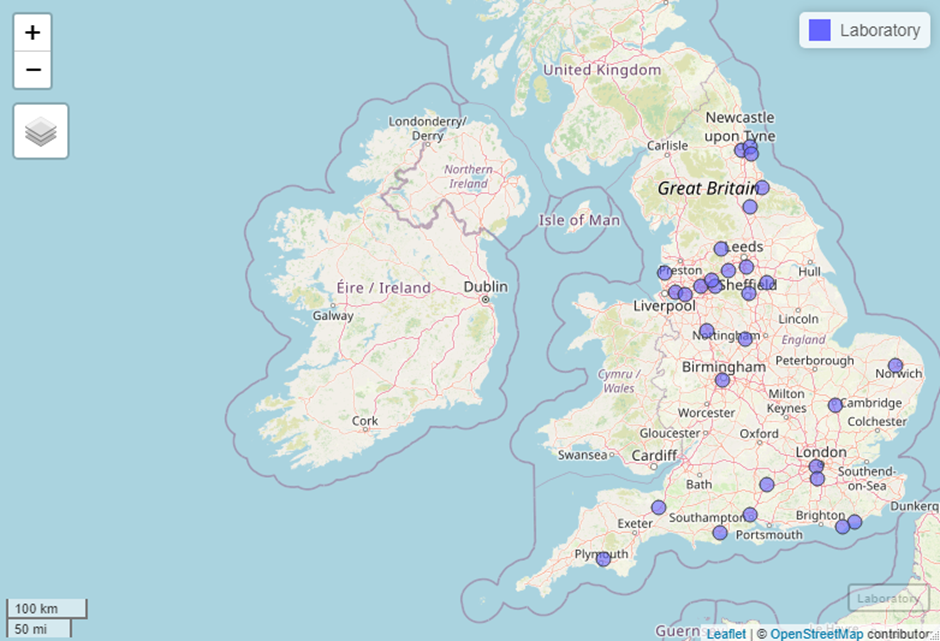


**Fig S2**. Laboratory locations across England included in this analysis.

Included laboratories were well distributed throughout England. Map created using Leaflet (RStudio).

|  | **N laboratories** | **N local laboratory alerts** | **Percent positive agreement** | **Gwet’s AC1** | **95% CI** |
| --- | --- | --- | --- | --- | --- |
| **LIMS1** | 7 | 274,286 | 0.966 | 0.951 | 0.950, 0.951 |
| **LIMS2** | 4 | 331,830 | 0.981 | 0.975 | 0.974, 0.975 |
| **LIMS3** | 1 | 75,392 | 0.976 | 0.970 | 0.970, 0.971 |

**Table S2**. Agreement within different laboratory information management system providers

Only a limited number of laboratories are included here since the LIMS provider was not always available or the laboratory currently uses its own unique LIMS. The proprietary names of LIMS providers have been anonymised. Abbreviations: **CliniSys**, Clinical Systems; **CSC**, Computer Science Corporation; **AC1**, agreement coefficient; **CI**, confidence interval.

|  | **Centrally generated alerts** | | | | |
| --- | --- | --- | --- | --- | --- |
|  | **0** | **1** | **2** | **3** | **Total** |
| **Local lab generated alerts after the first ever alert** |  |  |  |  |  |
| **0** |  |  |  |  |  |
| **Percent** | **79.48** | 1.65 | 0.45 | 0.37 | 81.96 |
| **Frequency** | **3,626,595** | 75,268 | 20,638 | 17,108 | 3,739,609 |
| **1** |  |  |  |  |  |
| **Percent** | 2.59 | **8.37** | 0.19 | 0.02 | 11.16 |
| **Frequency** | 118,393 | **381,709** | 8,554 | 730 | 509,386 |
| **2** |  |  |  |  |  |
| **Percent** | 0.49 | 0.39 | **2.45** | 0.05 | 3.39 |
| **Frequency** | 22,504 | 18,000 | **112,009** | 2,218 | 154,731 |
| **3** |  |  |  |  |  |
| **Percent** | 0.46 | 0.82 | 0.52 | **1.68** | 3.48 |
| **Frequency** | 21,195 | 37,240 | 23,847 | **76,628** | 158,910 |
| **Total** |  |  |  |  |  |
| **Percent** | 83.04 | 11.23 | 3.62 | 2.12 | **100.00** |
| **Frequency** | 3,788,687 | 512,217 | 165,048 | 96,684 | **4,562,636** |
|  |  |  |  |  |  |

**Table S3**. Matrix of numbers of lab versus central alerts for complete laboratories.

Laboratories with missing alert or SCr data for any month were excluded.

|  | **Centrally generated alerts** | | | | |
| --- | --- | --- | --- | --- | --- |
|  | **0** | **1** | **2** | **3** | **Total** |

| **Local lab generated alerts** |  |  |  |  |  |
| --- | --- | --- | --- | --- | --- |
| **1** |  |  |  |  |  |
| **Percent** | 10.63 | **49.93** | 1.35 | 0.65 | 62.56 |
| **Frequency** | 167,922 | **788,731** | 21,288 | 10,343 | 988,284 |
| **2** |  |  |  |  |  |
| **Percent** | 1.92 | 1.50 | **14.50** | 0.50 | 18.42 |
| **Frequency** | 30,252 | 23,711 | **229,065** | 7,970 | 290,998 |
| **3** |  |  |  |  |  |
| **Percent** | 1.93 | 4.02 | 2.85 | **10.21** | 19.01 |
| **Frequency** | 30,522 | 63,430 | 45,053 | **161,346** | 300,351 |
| **Total** |  |  |  |  |  |
| **Percent** | 14.48 | 55.45 | 18.70 | 11.37 | **100.00** |
| **Frequency** | 228,696 | 875,872 | 295,406 | 179,659 | **1,579,633** |
|  |  |  |  |  |  |

**Table S4**. Matrix of the numbers of laboratory-generated versus centrally derived alerts excluding AKI 0 alerts.

Zero alerts i.e., no AKI, had to be assumed if no AKI alert was received by the UKRR for that particular SCr value. Here, only AKI 1, 2 and 3 alerts generated by laboratories were compared to the central algorithm-generated alerts. Abbreviations: **lab**, laboratory.

|  | **N alerts** | **Percent positive agreement** | **Gwet’s AC1** | **95% CI** |
| --- | --- | --- | --- | --- |
| **Local lab alerts included assuming missing alerts were AKI 0 (Local lab AKI 0,1,2, and 3)** | 9,096,667 | 0.976 | 0.966 | 0.966, 0.966 |
| **Local lab alerts not generated on the basis that they were not received by the UKRR excluded (agreement only between local lab AKI 1,2, and 3)** | 1,579,633 | 0.920 | 0.825 | 0.825, 0.826 |

**Table S5**. Comparison of agreement coefficients for missing alerts versus assuming missing laboratory alerts were in fact AKI 0.

Coefficients were weighted. Abbreviations: **CI**, confidence interval; **AKI**, acute kidney injury; lab, laboratory; **UKRR**, UK Renal Registry; **AC**, agreement coefficient; **N**, number.

|  | **N alerts** | **Percent positive agreement** | **Gwet’s AC1** | **95% CI** |
| --- | --- | --- | --- | --- |
| **2015** | 125,824 | 0.968 | 0.956 | 0.956, 0.956 |
| **2016** | 224,363 | 0.973 | 0.964 | 0.963, 0.964 |
| **2017** | 302,863 | 0.976 | 0.967 | 0.967, 0.968 |
| **2018** | 346,131 | 0.978 | 0.970 | 0.970, 0.971 |
| **2019** | 357,731 | 0.978 | 0.970 | 0.969, 0.970 |
| **2020** | 222,721 | 0.972 | 0.959 | 0.959, 0.959 |

**Table S6**. Agreement over time, based on the year of laboratory-generated alert.

The deadline for algorithm implementation was March 2015 and data extraction was up until September 2020. Not all laboratories activated timeously. Abbreviations: **AC**, agreement coefficient; **CI**, confidence interval; **N**, number.

| **Quartile** | **Median SCr, μmol/L** | **Percent positive agreement** | **Gwet’s AC1** | **95% CI** |
| --- | --- | --- | --- | --- |
| **1** | 42 | 0.982 | 0.976 | 0.976, 0.976 |
| **2** | 65 | 0.990 | 0.986 | 0.986, 0.986 |
| **3** | 92 | 0.983 | 0.975 | 0.975, 0.975 |
| **4** | 164 | 0.927 | 0.877 | 0.876, 0.877 |

**Table S7**. Agreement across quartiles of baseline SCr (as determined by the central algorithm).

Abbreviations: **SCr**, serum creatinine; **AC**, agreement coefficient; **CI**, confidence interval.

| **Quintile**  **(Age range, years)** | **Median age, years** | **Percent positive agreement** | **Gwet’s AC1** | **95% CI** |
| --- | --- | --- | --- | --- |
| **1**  **(18 – 55.9)** | 45 | 0.922 | 0.831 | 0.829, 0.832 |
| **2**  **(56 – 68.9)** | 63 | 0.916 | 0.811 | 0.810, 0.813 |
| **3**  **(69 – 76.9)** | 72 | 0.915 | 0.810 | 0.808, 0.811 |
| **4**  **(77 – 83.9)** | 80 | 0.918 | 0.823 | 0.821, 0.825 |
| **5**  **(84 – 99)** | 88 | 0.927 | 0.852 | 0.851, 0.853 |

**Table S8**. Agreement across quintiles of age.

Coefficients are reported to 3 decimal places as the confidence intervals are narrow. Abbreviations: **CI**, confidence interval.
